# {5,5′-Dimeth­oxy-2,2′-[2,2-dimethyl­propane-1,3-diylbis(nitrilo­methanylyl­idene)]diphenolato}palladium(II)

**DOI:** 10.1107/S1600536812013128

**Published:** 2012-03-31

**Authors:** Siti Kamilah Che Soh, Mustaffa Shamsuddin, Mohd Mustaqim Rosli, Hoong-Kun Fun

**Affiliations:** a Department of Chemistry, Faculty of Science, Universiti Teknologi Malaysia, 81310 UTM Skudai, Johor, Malaysia; b Department of Chemical Sciences, Faculty of Science and Technology, Universiti Malaysia Terengganu, 21030 Kuala Terengganu, Terengganu, Malaysia; c Ibnu Sina Institute for Fundamental Science Studies, Universiti Teknologi Malaysia, 81310 UTM Skudai, Johor, Malaysia; d X-ray Crystallography Unit, School of Physics, Universiti Sains Malaysia, 11800 USM, Penang, Malaysia

## Abstract

In the title compound, [Pd(C_21_H_24_N_2_O_4_)], the complete mol­ecule is generated by crystallographic mirror symmetry with the Pd and three C atoms lying on the mirror plane. The Pd—O and Pd—N distances are 1.9932 (6) and 2.0029 (7) Å, respectively. The dihedral angle between two benzene rings of the ligand is 79.21 (4)°. In the crystal, C—H⋯O hydrogen bonds link the mol­ecules into layers parallel to the *ab* plane. These planes are further connected by C—H⋯O inter­actions, forming a three-dimensional network.

## Related literature

For related structures, see: Wan Nazihah Wan Ibrahim *et al.* (2008 ▶); Montazerozohori *et al.* (2009 ▶). For background to applications of palladium(II) complexes, see: Gupta *et al.* (2009 ▶); Lu *et al.* (2010 ▶); He & Cai (2011 ▶); Garoufis *et al.* (2008 ▶); Kumar *et al.* (2009 ▶); Islam *et al.* (2011 ▶). For the stability of the temperature controller used in the data collection, see: Cosier & Glazer (1986 ▶). 
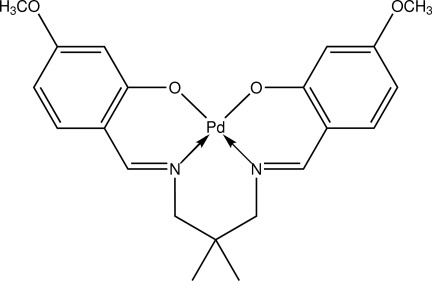


## Experimental

### 

#### Crystal data

[Pd(C_21_H_24_N_2_O_4_)]
*M* *_r_* = 474.82
Orthorhombic, 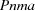

*a* = 11.5470 (4) Å
*b* = 20.9656 (7) Å
*c* = 7.8730 (3) Å
*V* = 1905.97 (12) Å^3^
*Z* = 4
Mo *K*α radiation
μ = 1.00 mm^−1^
*T* = 100 K
0.27 × 0.24 × 0.18 mm

#### Data collection

Bruker APEX DUO CCD area-detector diffractometer
Absorption correction: multi-scan (*SADABS*; Bruker, 2009 ▶) *T*_min_ = 0.773, *T*_max_ = 0.839
30756 measured reflections
4296 independent reflections
4134 reflections with *I* > 2σ(*I*)
*R*_int_ = 0.020

#### Refinement

*R*[*F* ^2^ > 2σ(*F* ^2^)] = 0.016
*wR*(*F* ^2^) = 0.043
*S* = 1.13
4296 reflections
134 parameters
H-atom parameters constrained
Δρ_max_ = 0.50 e Å^−3^
Δρ_min_ = −0.62 e Å^−3^

### 

Data collection: *APEX2* (Bruker, 2009 ▶); cell refinement: *SAINT* (Bruker, 2009 ▶); data reduction: *SAINT*; program(s) used to solve structure: *SHELXTL* (Sheldrick, 2008 ▶); program(s) used to refine structure: *SHELXTL*; molecular graphics: *SHELXTL*; software used to prepare material for publication: *SHELXTL* and *PLATON* (Spek, 2009 ▶).

## Supplementary Material

Crystal structure: contains datablock(s) I, global. DOI: 10.1107/S1600536812013128/kj2196sup1.cif

Structure factors: contains datablock(s) I. DOI: 10.1107/S1600536812013128/kj2196Isup2.hkl

Additional supplementary materials:  crystallographic information; 3D view; checkCIF report

## Figures and Tables

**Table 1:** Hydrogen-bond geometry (Å, °)

*D*—H⋯*A*	*D*—H	H⋯*A*	*D*⋯*A*	*D*—H⋯*A*
C8—H8*A*⋯O1^i^	0.99	2.37	3.3355 (10)	166
C12—H12*A*⋯O2^ii^	0.98	2.58	3.3719 (12)	138

Symmetry codes: (i) 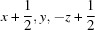; (ii) 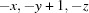.

**Fig. 1.**
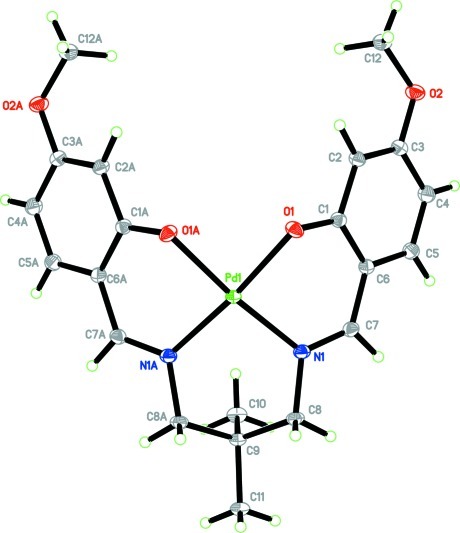
The molecular structure, showing 50% probability displacement ellipsoids. Hydrogen atoms are shown as spheres of arbitrary radius.

**Fig. 2.**
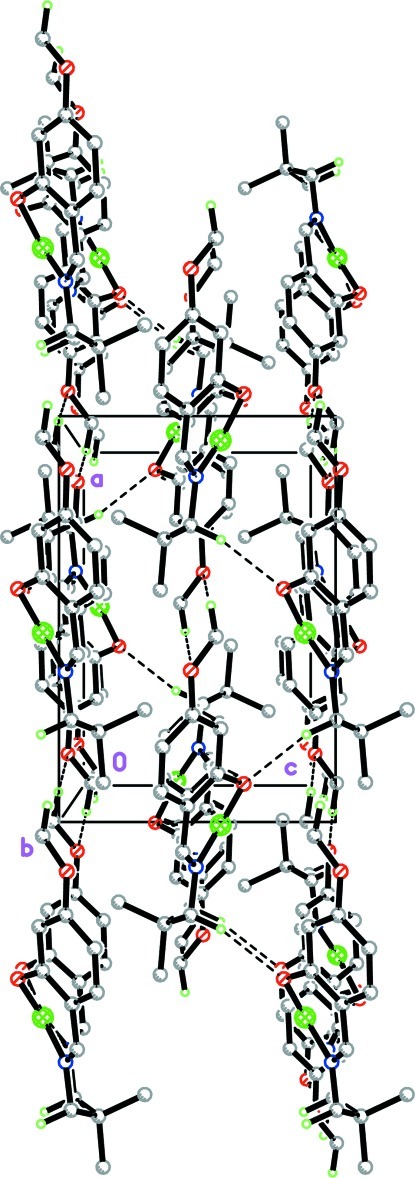
The crystal packing of (I). Dashed lines indicate hydrogen bonds. H atoms not involved in the hydrogen bond interactions have been omitted for clarity.

## supplementary crystallographic information

### Comment

Palladium(II)-Schiff base complexes have found extensive application in catalysis (Gupta *et al.*, 2009; Lu *et al.*, 2010; He and Cai 2011) and biological activities (Garoufis *et al.*, 2008). In particular, they are efficient and powerful phosphine-free catalysts for the formation of new C-C bonds in organic syntheses (Kumar *et al.*, 2009; Islam *et al.*, 2011). The title square-planar complex is analogous to the previously reported complex {2,2'-[(2,2 dimethylpropane-1,3-diyl)-bis(nitrilomethylidyne)]diphenolato}-palladium(II) ethanol hemisolvate (Wan Nazihah Wan Ibrahim *et al.*, 2008) in terms of geometry around the central palladium atom.

In the title compound, the complete molecule (Fig. 1) is generated by crystallographic mirror symmetry with the Pd1, C9, C10 and C11 atoms lying on the mirror plane. All parameters are within normal ranges and comparable with the related structures (Wan Nazihah Wan Ibrahim *et al.*, 2008). The Pd—O and Pd—N distances are 1.9932 (6)Å and 2.0029 (7)Å respectively. The dihedral angle between two benzene ring (C1-C6 & C1A-C6A) is 79.21 (4)°.

In the crystal packing, the molecules are linked by C8—H8A···O1^i^ intermolecular into layers parallel to the ab-plane. These planes are further connected by C12—H12A···O2^ii^ to form a three-dimensional network (Table 1 & Fig. 2).

### Experimental

The complex obtained was synthesized by dissolving the *N*,*N*'-bis(4-methoxy-salicylidene)-2,2-dimethylpropane-1,3-diamine ligand (0.2000 g, 0.54 mmol) in dry acetonitrile (10 ml) in a three necked round bottom flask. Palladium(II) acetate (0.1212 g, 0.54 mmol) which was dissolved separately in dry acetonitrile (10 ml) was then added into the flask containing the ligand solution. The mixture was stirred and refluxed under N_2_ gas atmosphere for 3 h. The yellow solid formed was separated by vacuum filtration, washed with cold acetonitrile and allowed to dry *in vacuo*. The solid product was then recrystallized from a mixture of dicholoromethane/methanol (1:1 *v*/*v*). Slow evaporation of the solvent at room temperature over several days gave yellow crystals (yield: 78%). Melting point: 596 K-598 K.

### Refinement

All H atoms attached to C atoms were fixed geometrically and refined as riding with C—H = 0.95–0.99 Å and *U*_iso_(H) = 1.2*U*_eq_(C) or 1.5*U*_eq_(*C*-methyl). A rotating group model was applied to the methyl group.

### Crystal data

**Table:**

[Pd(C_21_H_24_N_2_O_4_)]	*F*(000) = 968
*M**_r_* = 474.82	*D*_x_ = 1.655 Mg m^−^^3^
Orthorhombic, *P**n**m**a*	Mo *K*α radiation, λ = 0.71073 Å
Hall symbol: -P 2ac 2n	Cell parameters from 9824 reflections
*a* = 11.5470 (4) Å	θ = 3.3–35.1°
*b* = 20.9656 (7) Å	µ = 1.00 mm^−^^1^
*c* = 7.8730 (3) Å	*T* = 100 K
*V* = 1905.97 (12) Å^3^	Block, yellow
*Z* = 4	0.27 × 0.24 × 0.18 mm

### Data collection

**Table:**

Bruker APEX DUO CCD area-detector diffractometer	4296 independent reflections
Radiation source: fine-focus sealed tube	4134 reflections with *I* > 2σ(*I*)
Graphite monochromator	*R*_int_ = 0.020
φ and ω scans	θ_max_ = 35.1°, θ_min_ = 2.8°
Absorption correction: multi-scan (*SADABS*; Bruker, 2009)	*h* = −18→18
*T*_min_ = 0.773, *T*_max_ = 0.839	*k* = −33→33
30756 measured reflections	*l* = −8→12

### Refinement

**Table:**

Refinement on *F*^2^	Primary atom site location: structure-invariant direct methods
Least-squares matrix: full	Secondary atom site location: difference Fourier map
*R*[*F*^2^ > 2σ(*F*^2^)] = 0.016	Hydrogen site location: inferred from neighbouring sites
*wR*(*F*^2^) = 0.043	H-atom parameters constrained
*S* = 1.13	*w* = 1/[σ^2^(*F*_o_^2^) + (0.0183*P*)^2^ + 0.777*P*] where *P* = (*F*_o_^2^ + 2*F*_c_^2^)/3
4296 reflections	(Δ/σ)_max_ = 0.003
134 parameters	Δρ_max_ = 0.50 e Å^−^^3^
0 restraints	Δρ_min_ = −0.62 e Å^−^^3^

### Special details

**Table:**

Experimental. The crystal was placed in the cold stream of an Oxford Cryosystems Cobra open-flow nitrogen cryostat (Cosier & Glazer, 1986) operating at 100.0 (1) K.
Geometry. All e.s.d.'s (except the e.s.d. in the dihedral angle between two l.s. planes) are estimated using the full covariance matrix. The cell e.s.d.'s are taken into account individually in the estimation of e.s.d.'s in distances, angles and torsion angles; correlations between e.s.d.'s in cell parameters are only used when they are defined by crystal symmetry. An approximate (isotropic) treatment of cell e.s.d.'s is used for estimating e.s.d.'s involving l.s. planes.
Refinement. Refinement of *F*^2^ against ALL reflections. The weighted *R*-factor *wR* and goodness of fit *S* are based on *F*^2^, conventional *R*-factors *R* are based on *F*, with *F* set to zero for negative *F*^2^. The threshold expression of *F*^2^ > σ(*F*^2^) is used only for calculating *R*-factors(gt) *etc*. and is not relevant to the choice of reflections for refinement. *R*-factors based on *F*^2^ are statistically about twice as large as those based on *F*, and *R*- factors based on ALL data will be even larger.

### Fractional atomic coordinates and isotropic or equivalent isotropic displacement parameters (Å^2^)

**Table:**

	*x*	*y*	*z*	*U*_iso_*/*U*_eq_	
Pd1	0.535617 (6)	0.2500	0.088830 (10)	0.00982 (2)	
O1	0.42276 (5)	0.31425 (3)	0.17501 (8)	0.01411 (10)	
O2	0.12833 (5)	0.45403 (3)	−0.02329 (9)	0.01723 (11)	
N1	0.63494 (6)	0.31998 (3)	−0.00483 (9)	0.01240 (11)	
C1	0.39331 (7)	0.36205 (4)	0.07712 (9)	0.01203 (11)	
C2	0.27757 (7)	0.38427 (4)	0.08646 (10)	0.01310 (12)	
H2A	0.2251	0.3651	0.1642	0.016*	
C3	0.24037 (7)	0.43360 (4)	−0.01673 (10)	0.01370 (12)	
C4	0.31792 (7)	0.46562 (4)	−0.12613 (11)	0.01645 (13)	
H4A	0.2924	0.5004	−0.1939	0.020*	
C5	0.43136 (7)	0.44543 (4)	−0.13264 (11)	0.01566 (13)	
H5A	0.4844	0.4675	−0.2038	0.019*	
C6	0.47127 (6)	0.39285 (4)	−0.03651 (10)	0.01282 (12)	
C7	0.59049 (7)	0.37365 (4)	−0.05364 (10)	0.01349 (12)	
H7A	0.6415	0.4033	−0.1059	0.016*	
C8	0.76027 (6)	0.31049 (4)	−0.02515 (11)	0.01447 (13)	
H8A	0.7964	0.3083	0.0887	0.017*	
H8B	0.7932	0.3479	−0.0847	0.017*	
C9	0.79183 (9)	0.2500	−0.12444 (14)	0.01195 (16)	
C10	0.73574 (10)	0.2500	−0.29972 (15)	0.01630 (19)	
H10A	0.7594	0.2874	−0.3609	0.024*	
H10B	0.6530	0.2500	−0.2876	0.024*	
C11	0.92430 (10)	0.2500	−0.14109 (16)	0.01623 (19)	
H11A	0.9487	0.2874	−0.2017	0.024*	
H11B	0.9584	0.2500	−0.0299	0.024*	
C12	0.04407 (7)	0.41728 (5)	0.06862 (13)	0.01862 (15)	
H12A	−0.0332	0.4353	0.0496	0.028*	
H12B	0.0622	0.4185	0.1902	0.028*	
H12C	0.0457	0.3730	0.0288	0.028*	

### Atomic displacement parameters (Å^2^)

**Table:**

	*U*^11^	*U*^22^	*U*^33^	*U*^12^	*U*^13^	*U*^23^
Pd1	0.00822 (4)	0.01192 (4)	0.00933 (4)	0.000	0.00093 (2)	0.000
O1	0.0140 (2)	0.0151 (2)	0.0133 (2)	0.00348 (18)	0.00348 (19)	0.00199 (19)
O2	0.0125 (2)	0.0189 (3)	0.0203 (3)	0.0032 (2)	0.0008 (2)	0.0040 (2)
N1	0.0097 (2)	0.0144 (3)	0.0131 (3)	−0.0006 (2)	0.0015 (2)	−0.0015 (2)
C1	0.0122 (3)	0.0127 (3)	0.0112 (3)	0.0005 (2)	0.0008 (2)	−0.0011 (2)
C2	0.0122 (3)	0.0141 (3)	0.0130 (3)	0.0013 (2)	0.0015 (2)	0.0002 (2)
C3	0.0129 (3)	0.0144 (3)	0.0137 (3)	0.0016 (2)	−0.0001 (2)	−0.0007 (2)
C4	0.0165 (3)	0.0157 (3)	0.0172 (3)	0.0016 (2)	0.0013 (3)	0.0036 (3)
C5	0.0163 (3)	0.0145 (3)	0.0162 (3)	0.0001 (2)	0.0027 (3)	0.0021 (2)
C6	0.0121 (3)	0.0128 (3)	0.0135 (3)	0.0000 (2)	0.0017 (2)	0.0001 (2)
C7	0.0126 (3)	0.0138 (3)	0.0140 (3)	−0.0013 (2)	0.0020 (2)	−0.0007 (2)
C8	0.0089 (3)	0.0173 (3)	0.0171 (3)	−0.0012 (2)	0.0013 (2)	−0.0035 (3)
C9	0.0088 (4)	0.0152 (4)	0.0119 (4)	0.000	0.0012 (3)	0.000
C10	0.0142 (4)	0.0232 (5)	0.0115 (4)	0.000	0.0000 (3)	0.000
C11	0.0097 (4)	0.0200 (5)	0.0189 (5)	0.000	0.0025 (4)	0.000
C12	0.0132 (3)	0.0195 (4)	0.0232 (4)	0.0009 (3)	0.0012 (3)	0.0018 (3)

### Geometric parameters (Å, º)

**Table:**

Pd1—O1^i^	1.9932 (6)	C5—H5A	0.9500
Pd1—O1	1.9932 (6)	C6—C7	1.4406 (11)
Pd1—N1^i^	2.0029 (7)	C7—H7A	0.9500
Pd1—N1	2.0029 (7)	C8—C9	1.5337 (10)
O1—C1	1.3092 (9)	C8—H8A	0.9900
O2—C3	1.3637 (10)	C8—H8B	0.9900
O2—C12	1.4366 (11)	C9—C10	1.5244 (16)
N1—C7	1.2951 (10)	C9—C8^i^	1.5336 (10)
N1—C8	1.4695 (10)	C9—C11	1.5353 (15)
C1—C2	1.4172 (11)	C10—H10A	0.9600
C1—C6	1.4239 (11)	C10—H10B	0.9600
C2—C3	1.3835 (11)	C11—H11A	0.9599
C2—H2A	0.9500	C11—H11B	0.9600
C3—C4	1.4122 (12)	C12—H12A	0.9800
C4—C5	1.3776 (12)	C12—H12B	0.9800
C4—H4A	0.9500	C12—H12C	0.9800
C5—C6	1.4144 (11)		
			
O1^i^—Pd1—O1	85.03 (3)	C1—C6—C7	122.43 (7)
O1^i^—Pd1—N1^i^	90.27 (3)	N1—C7—C6	126.41 (7)
O1—Pd1—N1^i^	174.10 (3)	N1—C7—H7A	116.8
O1^i^—Pd1—N1	174.10 (3)	C6—C7—H7A	116.8
O1—Pd1—N1	90.27 (3)	N1—C8—C9	113.67 (7)
N1^i^—Pd1—N1	94.20 (4)	N1—C8—H8A	108.8
C1—O1—Pd1	119.13 (5)	C9—C8—H8A	108.8
C3—O2—C12	117.06 (7)	N1—C8—H8B	108.8
C7—N1—C8	118.40 (7)	C9—C8—H8B	108.8
C7—N1—Pd1	121.25 (5)	H8A—C8—H8B	107.7
C8—N1—Pd1	120.32 (5)	C10—C9—C8^i^	111.13 (6)
O1—C1—C2	117.78 (7)	C10—C9—C8	111.13 (6)
O1—C1—C6	123.55 (7)	C8^i^—C9—C8	111.56 (9)
C2—C1—C6	118.67 (7)	C10—C9—C11	110.24 (9)
C3—C2—C1	120.53 (7)	C8^i^—C9—C11	106.28 (6)
C3—C2—H2A	119.7	C8—C9—C11	106.28 (6)
C1—C2—H2A	119.7	C9—C10—H10A	109.5
O2—C3—C2	123.47 (7)	C9—C10—H10B	109.5
O2—C3—C4	115.41 (7)	H10A—C10—H10B	109.5
C2—C3—C4	121.11 (7)	C9—C11—H11A	109.6
C5—C4—C3	118.66 (7)	C9—C11—H11B	109.3
C5—C4—H4A	120.7	H11A—C11—H11B	109.5
C3—C4—H4A	120.7	O2—C12—H12A	109.5
C4—C5—C6	121.96 (7)	O2—C12—H12B	109.5
C4—C5—H5A	119.0	H12A—C12—H12B	109.5
C6—C5—H5A	119.0	O2—C12—H12C	109.5
C5—C6—C1	118.93 (7)	H12A—C12—H12C	109.5
C5—C6—C7	118.62 (7)	H12B—C12—H12C	109.5
			
O1^i^—Pd1—O1—C1	−132.69 (5)	C3—C4—C5—C6	1.59 (13)
N1—Pd1—O1—C1	43.75 (6)	C4—C5—C6—C1	−3.42 (13)
O1—Pd1—N1—C7	−29.00 (6)	C4—C5—C6—C7	178.41 (8)
N1^i^—Pd1—N1—C7	147.14 (5)	O1—C1—C6—C5	−177.99 (7)
O1—Pd1—N1—C8	153.13 (6)	C2—C1—C6—C5	1.59 (11)
N1^i^—Pd1—N1—C8	−30.72 (7)	O1—C1—C6—C7	0.11 (12)
Pd1—O1—C1—C2	144.53 (6)	C2—C1—C6—C7	179.69 (7)
Pd1—O1—C1—C6	−35.88 (10)	C8—N1—C7—C6	−176.76 (8)
O1—C1—C2—C3	−178.41 (7)	Pd1—N1—C7—C6	5.33 (11)
C6—C1—C2—C3	1.98 (11)	C5—C6—C7—N1	−164.51 (8)
C12—O2—C3—C2	−6.96 (12)	C1—C6—C7—N1	17.38 (13)
C12—O2—C3—C4	171.91 (8)	C7—N1—C8—C9	−125.73 (8)
C1—C2—C3—O2	174.91 (7)	Pd1—N1—C8—C9	52.19 (9)
C1—C2—C3—C4	−3.90 (12)	N1—C8—C9—C10	57.06 (10)
O2—C3—C4—C5	−176.79 (8)	N1—C8—C9—C8^i^	−67.56 (11)
C2—C3—C4—C5	2.11 (13)	N1—C8—C9—C11	177.01 (7)

Symmetry code: (i) *x*, −*y*+1/2, *z*.

### Hydrogen-bond geometry (Å, º)

**Table:**

*D*—H···*A*	*D*—H	H···*A*	*D*···*A*	*D*—H···*A*
C8—H8*A*···O1^ii^	0.99	2.37	3.3355 (10)	166
C12—H12*A*···O2^iii^	0.98	2.58	3.3719 (12)	138

Symmetry codes: (ii) *x*+1/2, *y*, −*z*+1/2; (iii) −*x*, −*y*+1, −*z*.
